# Report on advances for pediatricians in 2018: allergy, cardiology, critical care, endocrinology, hereditary metabolic diseases, gastroenterology, infectious diseases, neonatology, nutrition, respiratory tract disorders and surgery

**DOI:** 10.1186/s13052-019-0727-6

**Published:** 2019-10-16

**Authors:** Carlo Caffarelli, Francesca Santamaria, Carla Mastrorilli, Angelica Santoro, Brunella Iovane, Maddalena Petraroli, Valeria Gaeta, Rosita Di Pinto, Melissa Borrelli, Sergio Bernasconi, Giovanni Corsello

**Affiliations:** 1 Clinica Pediatrica, Department of Medicine and Surgery, Azienda Ospedaliera-Universitaria, University of Parma, Via Gramsci 14, Parma, Italy; 2 Department of Translational Medical Sciences, Federico II University, Naples, Italy; 3 UO Pediatria e Pronto Soccorso, Azienda Ospedaliero-Universitaria Consorziale Policlinico Pediatric Hospital Giovanni XXIII, Bari, Italy; 4 UOC Pediatria Generale e d’Urgenza, Department of Medicine and Surgery, Azienda Ospedaliera-Universitaria, University of Parma, Parma, Italy; 5 Pediatrics Honorary Member University Faculty, G D’Annunzio University of Chieti-Pescara, Chieti, Italy; 6 Department of Sciences for Health Promotion and Mother and Child Care “G. D’Alessandro”, University of Palermo, Palermo, Italy

**Keywords:** Allergy, Cardiology, Children, Critical care, Endocrinology, Hereditary metabolic diseases, Gastroenterology, Infectious diseases, Neonatology, Nutrition, Respiratory tract disorders, Surgery

## Abstract

This review reported notable advances in pediatrics that have been published in 2018. We have highlighted progresses in allergy, cardiology, critical care, endocrinology, hereditary metabolic diseases, gastroenterology, infectious diseases, neonatology, nutrition, respiratory tract disorders and surgery. Many studies have informed on epidemiologic observations. Promising outcomes in prevention, diagnosis and treatment have been reported. We think that advances realized in 2018 can now be utilized to ameliorate patient care.

## Background

Numerous impressive papers were published in the Italian Journal of Pediatrics in 2018. This review presents the most accessed articles published over the last year. The most recent relevant updates on allergy, cardiology, critical care, endocrinology, hereditary metabolic diseases, gastroenterology, infectious diseases, neonatology, nutrition, respiratory tract disorders and surgery in childhood have been highlighted. Information are also provided to contextualize each article. We believe that this review will help readers to understand novel advances in pediatrics and to provide the best patient care.

## Review

### Allergy and respiratory tract disorders

#### 1- risk factors for atopic diseases 2-acute asthma treatment 3-provocation challenge to drugs

Asthma, food allergy [1] and atopic diseases are common in Western countries, particularly in immigrants. Along this line, prevalence of allergen sensitization was higher in adopted children from developing countries with lower prevalence of atopy than in non-adopted children in Italian population [2]. These findings underline that environmental factors can predispose to the development of atopic disease in immigrants, even if sensitization is not always associated with clinical hypersensitivity reactions [3]. A key environmental risk factor for atopy in immigrants is bad housing [4]. Additional studies are needed to identify immigrants at risk for atopic diseases and which factors are involved with aim of identifying preventive intervention.

A guideline for the management of acute asthma attack in childhood was issued in the past year [5]. A multidisciplinary panel undertook a comprehensive review of the published evidence. The level of evidences and the strength of the recommendations were provided according to pre-established standards. Criteria for assessing severity of asthma attack were given. Regarding treatment, inhaled salbutamol was recommended as the first-line treatment and systemic steroids should be added when necessary. Epinephrine injections do not control symptoms better than β2 agonist as well as inhaled steroid compared to systemic steroids. When treatment is not effective, iv salbutamol or iv aminophylline should be given. MgSO4 or a helium-oxygen mixture (70%: 30%) could be used in children who do not respond. Leukotriene modifiers are unhelpful. Biomarkers for starting or discontinuing treatment during asthma attack are warranted. Among them, levels of pH in exhaled breath condensate may be helpful [6]. There is some evidence of inflamed airway in children with atopic eczema who do not suffer from asthma [7]. It may be possible that the detection of inflammatory markers may be useful for identifying who are at risk of asthma exacerbation. Controllers, mainly inhaled corticosteroids, are used for keeping allergic asthma under control, preventing asthma attacks, enhancing quality of life and bringing to an end the decay of lung function. Studies continue to highlight the role of aeroallergens [8] and food allergens [9] in eliciting asthma attacks. So, allergen-specific immunotherapy (AIT) should be considered in children with allergic asthma when daily pharmacotherapy does not achieve a satisfactory asthma control [10]. AIT is recommended in children with asthma due to house dust mite to reduce symptoms and drug consumption, Both subcutaneous and sublingual AIT have been also shown to ameliorate allergic rhinitis or rhino-conjunctivitis with/without allergic asthma to pollens such as grass or birch pollen and prevent asthma onset in children [11]. Of note, clinical benefits are maintained for years following AIT cessation. Furthermore, there are some evidence that sublingual AIT to mites can be of benefit not only in children with atopic respiratory diseases but also in children with atopic eczema [12]. Provocation challenge to antibiotics and non-steroidal anti-inflammatory drugs is often necessary to diagnose IgE-mediated and delayed reactions and to remove drug allergy label. A document reporting indication, contraindication and protocols for provocation test that are extremely useful in practice, has been provided [13]. This paper reflects current knowledge on the issue. It updates previous position papers taking into account expanding knowledge and experience in addressing diagnosis of drug allergy [14–17].

### Cardiology

#### 1- Kawasaki disease

Kawasaki disease (KD) is an acute systemic vasculitis, that may involve small and medium vessels in each organ and apparatus with a multi-factorial etiology, and affects children < 5 years of age in 80% of cases and it is generally attributed to an amplified immunologic response to unknown environmental or infectious triggers [18] among genetically susceptible children [19]. Marchesi A et al. [20, 21] representing the Italian Society of Pediatrics provided recommendations on recognition and management of KD. The course of KD can be distinguished in three phases: acute phase, with fever, bilateral conjunctivitis without exudate, erythema of oral mucosa, erythema of the palms and soles, polymorphic skin rash, cervical lymphadenopathy lasting 2 weeks; subacute phase, characterized by digital skin desquamation, thrombocytosis, possible coronary artery aneurysms (CAA) (third and fourth week); convalescence phase, normal inflammatory indexes, persisting endothelial dysfunction (fifth to eighth week). The treatment of acute phase of KD aims at reducing the inflammation and decreasing the risk of CAA with high-dose intravenous immunoglobulin (IVIG) and acetylsalicylic acid [22]. However, 10–20% of patients do not respond to initial IVIG, and additional therapies are recommended. Complications of KD can be distinguished in: resistant forms; recurrent forms; cardiovascular/systemic complications. Therapies of resistant forms have been revised particularly concerning corticosteroids and biological drugs, of coronary artery thrombosis and cardiovascular complication with anticoagulant drugs and anti-platelets [23]. Moreover, the follow-up and the prevention techniques have been pointed out, according to cardiovascular risk classes with short- and long-term recommendations. Regarding cardiovascular complications, myocarditis may be involved in the acute phase without differences between patients affected or not by CAA [24]. Clinical signs and symptoms of KD vary from asymptomatic to hyperdynamic precordium, hemodynamic instability and ventricular dysfunction that may necessitate fluid resuscitation and vasoactive treatment. Among cardiovascular events, also myocarditis may occur and its diagnosis needs to be sustained by imaging (nuclear imaging, echocardiography, magnetic resonance), serum biomarkers and biopsy. Long-term clinical consequences of myocardial inflammation, although rare, can appear in the setting of CAA. Echocardiography represents the gold-standard to identify CAA during the acute phase of KD [25]. Recently it has been adopted an international coronary artery z score with the measurement of the coronary artery diameter and the body surface area, that permits to define ectasia, slight expansion (diameter < 5 mm), moderate dilatation (5–8 mm), and giant aneurysm (> 8 mm) [22]. Echocardiography should be performed at the diagnosis and after 1–2 weeks and 4–6 weeks of the treatment, in uncomplicated cases without important coronary artery involvement [25]. Standardized imaging protocols are needed for diagnosis and follow-up of cardiovascular events in KD.

### Critical care

#### 1 - pediatric head injury 2 - vitamin K deficiency

In developed countries the most common causes for presentation to the emergency department (ED) among children are injuries, that represent a frequent source of disability and death. The Italian Society of Pediatric Emergency Medicine has published a guideline on pediatric head injury to help ED physicians in treating children with a blunt head trauma [26]. The initial assessment of stabilization in children with severe head trauma include the ABCDE approach: Airway with cervical spine protection; Breathing; Circulation (hemorrhage control); Disability (neurologic status); Exposure (undress) and Environment (temperature control). Cchildren with head trauma should be monitored within the first 24 h of their injury. The diagnosis of clinically important traumatic brain injury includes the age appropriate PECARN algorithms to assist the decision to perform a head CT scan in children with a GCS < 14. In children with severe blunt head trauma and signs of raised intracranial pressure it should be administered hyperosmolar treatment with hypertonic saline [27]. Hhyperventilation, except those with signs of impending cerebral herniation, in which represents a life-saving action should be avoided.

The deficiency of vitamin K causes a disorder of hemostasis, called vitamin K deficiency bleeding (VKDB), that typically occurs in the postnatal period. Therefore, in order to prevent VKDB at birth all neonates receive vitamin K prophylaxis with 1 mg intramuscularly or 2 mg orally. Three forms can be distinguished: early VKDB, that occurs in the first 24 h of life; classic VKDB, from the first to 7 days after birth; late form, between the 2nd and the 6th weeks of life. Hemorrhagic manifestations are usually gastrointestinal and cutaneous, or additionally cerebral in late forms. Infants with late VKDB presented with neurological signs [28] or with bleeding of the skin at the vaccine site [29]. Both neonates received oral vitamin K prophylaxis at birth. However, oral route can be affected by different irregularities of absorption, such as absorption, unknown cholestasis, vomiting, setting. According to recent studies and international guidelines, intramuscular administration of vitamin K is clinically more effective than oral one especially in late forms, so it should be preferred at birth [30, 31]. The administration of oral 2 mg vitamin K at birth, repeated twice at 2–4 and 6–8 weeks of age, should be limited to newborns whose parents decline intramuscular vitamin K [32]. Moreover, it has been proposed to increase the dose of vitamin K prophylaxis with a 6-fold increase of daily regimen, demonstrating a significant although modest reduction of intracranial VKDB in a Dutch nationwide survey [33]. Univocal recommendations about vitamin K administration at birth should be given.

### Endocrinology

#### 1 - vitamin D 2 - hypothyroidism 3- premature pubarche

In recent years, many studies have been published on the role that vitamin D plays in various physio-pathological conditions. This renewed interest arises from several factors: the reappearance of rickets especially in less privileged populations even after a prophylaxis applied for years that had made the disease disappear [34], the results of epidemiological studies in different Countries showing non optimal blood levels of 25 (OH) D in many childhood populations, even in those living in economically well-developed societies, and finally the discovery that the vitamin has a pleiotropic action and may therefore interfere in various biological mechanisms, not only on bone health.

However, the results obtained thus far have not made it possible to reach definitive and universally accepted conclusions.

Even the definition of optimal blood levels is a source of discussion among those who believe that a level of 20 ng/ml is sufficient for normal bone growth (although this figure is mainly acquired from the experience in adults) and those who deem necessary levels of at least 30 ng/ml considering the pleiotropic potential of the vitamin.

It is therefore useful that groups of pediatric specialists in various Countries carry out a careful review of literature, taking into account local realities, in order to suggest shared behaviors to family pediatricians.

In this sense, the recently published Italian Consensus [35] that recommends the dose of vitamin D in the various age groups is extremely useful, also considering recent dietary surveys which showed insufficient vitamin supplementation during the first years of life [36].

Among the possible extra skeletal actions of vitamin D numerous researches have tried to determine if there is a role in the pathogenesis of autism.

Low blood levels of 25-hydroxy vitamin D have been found in a group of children with autism spectrum disorder (ASD) living in Ahvaz, a city in the southwest of Iran [37]. This result is in agreement with a recent meta-analysis [35] which showed that serum 25(OH) D levels were significantly lower in children and adolescents with ASD than in controls.

The possible role of 25(OH) D in ASD pathogenesis has been matter of discussion for many years but no definitive conclusion or general agreement has been achieved. Epidemiological studies have shown a dramatically increased prevalence of ASD which (at least partially) has been ascribed to environmental factors.

In fact, there is a general agreement on the hypothesis that the interaction between genetic and environmental factors causes most ASD [38]. Among these environmental factors 25(OH) D may play a pivotal role through several mechanisms which have been extensively reviewed [38]. In particular, calcitriol (the activated vitamin D hormone) is considered a neurosteroid hormone capable of protecting brain tissue by reducing inflammatory cytokine levels.

The translation of experimental data into clinical practice is however open to different opinions.

The Authors of the Italian consensus on vitamin D [35] concluded that further research is needed before recommending routine vitamin D supplementation in children with ASD. However, some Authors conclude that although scientific data on the role of vitamin D in the prevention or treatment of autism is preliminary, this does not relieve practitioners from using all current available scientific evidence to make a risk benefit analysis of whether or not to treat or supplement their patients with vitamin D up to 40–60 ng/ml, levels recommended by the Endocrine Society [38].

A third paper published on the Italian Journal of Pediatrics drew attention to an aspect that has not been studied in depth. Doneray et al. concluded that maternal and neonatal vitamin D-binding protein (DBP) levels may lead to an incorrect low estimate of true Vitamin D concentrations [39]. The major circulating form of vitamin D is 25-hydroxyvitamin D (25(OH)D). In the circulation, vitamin D (like other steroid hormones) is tightly bound to a special carrier – vitamin D-binding protein (DBP). Smaller amounts are bound to blood proteins – albumin and lipoproteins. Only very tiny amounts of total vitamin D are free and potentially biologically active. Currently used vitamin D assays do not distinguish between the three forms of vitamin D – DBP-bound vitamin D, albumin-bound vitamin D, and free biologically active vitamin D. Sex steroids, in particular estrogens, stimulate the synthesis of DBP. This explains why total vitamin D concentrations are higher during pregnancy as compared to nonpregnant women, while the concentrations of free vitamin D remain similar in both groups of women [40]. The free hormone hypothesis postulates that only free 25(OH) D can enter cells. This hypothesis is supported by the observation that mice lacking DBP, and therefore with essentially undetectable 25(OH) D levels, do not show signs of vitamin D deficiency unless put on a vitamin D deficient diet. This hypothesis also applies to other protein bound lipophilic hormones including glucocorticoids, sex steroids, and thyroid hormones [41].

Further studies are therefore necessary to understand whether the dosage of binding protein and the free fraction of the vitamin can provide us with further information in terms of pathogenesis of various diseases and help to better define vitamin needs in the individual. The need to find validated and widely available laboratory methods that allow a correct determination of these parameters should also be considered.

Gallizzi et al. [42] reviewed the main clinical and metabolic abnormalities that might be observed in children with longstanding and untreated subclinical hypothyroidism (SH). They highlighted that there is no evidence of any neurocognitive outcome, except for the attention level. L-T4 treatment does not seem to be able to improve the attention problems. Linear growth, height, growth velocity or bone maturation, bone mineral homeostasis are not affected [43]. Hyperthyrotropinemia should be considered a consequence rather than the cause of obesity in overweight children with SH, supposing that this condition might play a key role in conditioning the risk of Metabolic Syndrome. Children with mild and untreated SH might have an increased cardiovascular risk because this condition is related to early changes in proatherogenic profile (increased concentrations in asymmetric dimethylarginine, marker of endothelial dysfunction) [44], particularly in obese children. SH should be treated [45] according to patient’s age, with TSH values> 10 uUI/ml, for progressively increasing TSH serum levels or according to the presence of Hashimoto Thyroiditis.

Many studies have been published on the higher risk of insulin resistance, obesity and later Metabolic Syndrome or PCOS in children with premature adrenarche, probably caused by the influence of adrenal androgens (DEA, DHEAS, 17OH pregnenolone, 17OH progesterone) on glucose homeostasis and on ovarian function [46, 47]. In order to detect predictive factors of premature pubarche, Cavarzere et al. published the first study analysing the relation between premature pubarche and the 17OHP level at newborn screening for CAH [48]. They found no correlation between elevated 17OHP levels at screening and the development of premature pubarche, excluding the utility of this value in predicting it.They recommended making all confirmatory tests in children with elevated 17OHP levels at screening only later on, if further symptoms appear. Among children with precocious pubarche ACTH test should be performed when ratio between bone age and statural age exceeds 1 and/or elevated androgen levels for age or cystic acne or other signs of systemic virilization, in order to identify also late onset CAH.

### Gastroenterology

#### 1 – functional gastrointestinal disorders; 2 – acute gastroenteritis

Abdominal pain is present in different functional gastrointestinal disorders (FGIDs), particularly in irritable bowel syndrome (IBS). The pathogenesis of abdominal pain in FGDIs remains unclear. Dietary intervention can play a role in non-pharmacological management of FGIDs. Taking into account that FGIDs are not associated with immunologically mediated reactions to foods [49] a diet free of common allergens usually fails and it has been abandoned. FODMAPs are poorly absorbed carbohydrates that are fermentable by colonic bacteria with gas production and increase osmotic action. In the last decade, a low fermentable oligosaccharides, disaccharides, monosaccharides, and polyol (FODMAPs) diet has been increasingly used for treating FGIDs [50–52]. Turco R et al. [53] showed that there is evidence that a low FODMAPs diet is of benefit in adults with IBS. In children, further studies are warranted to clarify the efficacy of a restricted diet in FODMAPs.

Acute gastroenteritis continues to be a significant focus of research since it is a common infectious disease in children with elevated mortality rates in developing countries [54]. Several approaches have been proposed for managing symptoms of acute gastroenteritis including probiotics [55], antidiarrheal agents, antiemetic, zinc [56]. In a randomized, case-controlled trial, natural molecular complex of tannins and flavonoids plus standard oral rehydration (SOR) was more effective than standard oral rehydration (SOR) alone in reducing number of stools after 24 h of treatment [57]. The treatment was well tolerated and safe in 30 children. Larger studies are required on this approach [58].

### Hereditary metabolic diseases

#### 1 – clinical features; 2 - mucopolysaccharidoses

When newborns and infants experience acute or recurrent symptoms after food ingestion, first of all immune-mediated adverse food reactions are considered in the list of the possible causes, but also non-immunological food reactions, motility disorders or anatomic abnormalities of the gastrointestinal tract, infections, systemic diseases, inherited metabolic disorders (IMDs) should be included in the differential diagnosis [59].

Maines et al. report clinical features and diagnostic aspects of the most common IMDs that may present with acute manifestations triggered by food intake [60]. Inherited metabolic disorders (IMDs) are a complex and heterogeneous group of rare monogenic disorders, usually resulting from a deficient activity in a single pathway of intermediary metabolism [61]. Each IMD is rare if considered alone, but the cumulative incidence is estimated 1:1500–5000 [62, 63]. Deficiencies of enzymes or transporters involved in the amino acid and/or protein metabolism may present acutely in the neonatal period or later in life with acute, intermittent or progressive symptoms. Catabolic states or increased protein intake are the most common triggers that provoke acute metabolic attacks with vomiting or feeding difficulties accompanied by lethargy or encephalopathy that may rapidly progress to coma, and liver failure [64]. Actually, for many IMDs (in particular, the distal urea cycle disorders, the organic acidurias, and the maple syrup urine disease) the risk of an acute presentation triggered by food is decreased thanks to the introduction of expanded newborn screening (NBS). Through few laboratory data (including ketones, ammonia and blood gas analysis) performed during an acute attack, it is possible to provide a first differential diagnosis of IMDs triggered by proteins. Intoxication may also be caused by toxic carbohydrate metabolites derived by exogenous intake of galactose and fructose, respectively [65, 66]. If galactosemia is the underlying disorder, breastfeeding or lactose-containing formula feeding during the first days of life can cause severe liver dysfunction, that may present with jaundice, hepatomegaly, hypoglycemia, coagulation disturbances, and gastrointestinal findings of poor feeding, vomiting and diarrhea. The onset of illness may be acute and fulminant and may often be confused with neonatal sepsis due to the *E. coli* infection [67]. The hereditary fructose intolerance usually presents at the time of weaning, when fruits and vegetables are introduced into the diet. Acute symptoms include gastrointestinal discomfort, feeding difficulties, vomiting, pallor, metabolic acidosis, hepatomegaly, hypoglycemia, restlessness, lethargy and shock. Generally, these patients are asymptomatic since they avoid foods containing fructose or any of its common precursors (such as sucrose and sorbitol), while prolonged fructose ingestion can ultimately lead to liver failure and renal dysfunction [65, 66]. In conclusion, while IMDs clinical suspicion remains essential because some IMD do not have still reliable markers for NBS and a false negative screening result may occur, on the other hand high clinical suspicion alone is not sufficient to exclude the disorder, therefore expanded NBS needs to be considered as a mandatory public health strategy [68]. Early diagnosis of several IMDs makes it possible to modify their course reducing morbidity and mortality. In a recent review Joseph et al. [69] have proposed a screening test of Hunter syndrome and of mucopolysaccharidosis type II. Smon et al. have proposed to use an expanded NBS approach that includes the next-generation sequencing (NGS) for confirming the clinical suspicion [70]. These authors concluded that not only NGS is valuable for explaining the abnormal metabolite concentrations in NBS by enabling the distinction between affected individuals and mere heterozygotes, but also that it improves the turnaround time of genetic analysis.

Mucopolysaccharidoses (MPS) are a heterogeneous group of inherited metabolic disorders, each associated with a deficiency of the enzymes involved in the glycosaminoglycans (GAGs) catabolism. GAGs accumulate in cells and tissues, with progressive damage in different organs that leads to premature death. The ear, nose, and throat (ENT) manifestations in MPS, due to the accumulation of GAGs in the head and neck region, are very common in the MPS population [71, 72]. They include protruding or depressed frontal bone, a depressed nasal bridge, wide nasal alae, thick lips, angled and hypoplastic mandible (micrognathia), macroglossia, distorted teeth, gingival hypertrophy, also restriction of the mouth opening, with moderate-to-severe adenotonsillary hypertrophy and thickening of the soft tissues in the laryngopharynx. Bianchi et al. [73] have investigated the role of otolaryngologists in the multidisciplinary approach to paediatric or adult MPS [74]. Respiratory disorders occur in all type of MPS since affected individuals classically have a number of anatomical features that predispose to airway dysfunction [75, 76]. Airway problems include obstructive sleep apnea (OSA), frequent respiratory infections, adenoid and tonsillar hypertrophy, irregular nasal septum, turbinate hypertrophy, macroglossia, thickened pharyngeal wall, laryngeal abnormalities, tracheomalacia, tracheal stenosis and short neck, dyspnea, restricted joint mobility, skeletal abnormalities, and increased mucus secretions in the upper and lower airways [77–81]. Chronic rhinosinusitis and chronic otitis media lead to hearing impairment [76]. Upper and lower respiratory tract infections are frequent. Respiratory disorders in MPS can be divided into airway abnormalities (extra thoracic and intrathoracic), and alterations in the respiratory and sleeping mechanics [82]. Extra thoracic anatomical abnormalities and GAGs infiltration of the nasopharyngeal, oropharyngeal, hypopharyngeal, and laryngeal tissues, predispose children with MPS to severe upper airway obstruction [83]. Intrathoracic airway obstruction is also a common complication, and it could be complicated by tracheobronchial abnormalities [82]. The trachea can be narrow, tortuous, or occluded by the accumulation of soft tissue [83]. Therefore, most MPS patients have stridor, dyspnea, retractions, cough, cyanosis, or difficulty with feeding [84], and the severity of respiratory dysfunction varies according to the MPS type [83]. The combined effect of these alterations in respiratory mechanics together with airway abnormalities may lead to irreversible and fatal respiratory failure.

All MPS types may have severe or attenuated presentations, this depending on the residual enzymatic activity found. Galimberti et al. have underlined the very early signs of the severe forms of MPS which should alert the paediatrician on their first appearance [85]. In the first 6 months of life, inguinal hernias, abnormally frequent respiratory infections, otitis, and organomegaly can be seen in MPS patients, particularly in MPS I (caused by a deficiency of the lysosomal hydrolase α-l-iduronidase). Moreover, from 6 to 12 months, these infants very often develop a gibbus due to the thoraco-lumbar kyphosis, present with mild hypotonia, and develop growth delay. Symptoms and signs suggestive of MPS, should promptly arouse suspicion of MPS in any infant or child presenting with one or more of these features. Referring these patients to a specialized centre will allow the recognition of these disorders at a very early stage. Kuiper et al. [86] have reported on attenuated forms of MPS, and concluded that many patients may remain apparently asymptomatic for years and are frequently diagnosed at an advanced stage, this confirming the relevant importance of new pilot newborn screening programs for MPS.

### Infectious diseases

#### 1 – human immunodeficiency virus; 2 - vaccination

Human immunodeficiency virus (HIV) develops very rapidly among infants and children. If untreated, half of children with HIV will die before their second birthday and one third before year one [87]. At the end of 2016, 2.1 million children younger than 15 years old were affected by HIV, and more than half of them were from sub-Saharan Africa. Undernutrition is a major public health problem in countries with high prevalence of HIV. Ethiopia is one of the sub-Saharan countries with the highest prevalence of HIV, and malnutrition is frequent [88]. The relationship between malnutrition and HIV is bidirectional: on one side malnutrition accelerates the progress of HIV infection, on the other, once HIV occurs, the patient’s nutritional status declines rapidly and leads to immune depletion and HIV progression [89]. In Ethiopia, although individuals are often dually impacted, the effect of undernutrition on the survival of HIV positive children treated with anti-retroviral therapy (ART) has not been well investigated. Alebel et al. conducted a 5-years study considering nutritional status as an independent predictor of survival in 390 HIV positive children treated with ART from Amhara Regional State Referral Hospitals, Ethiopia [90]. They found an overall mortality rate of 4.4 per 100 child-years and demonstrated that undernourished children had a lower survival time than well-nourished peers. Moreover, as well as severe stunting, low hemoglobin level, CD4 cell count or percent below the threshold, severe wasting and advanced disease stage (III and IV) were found to be good predictors of mortality. This finding is consistent with a study conducted in Northern Ethiopia on a cohort of 149 HIV-1-infected children that showed that early mortality (death < 18 months after ART initiation) was higher than late mortality (death ≥18 months after ART initiation) and that low baseline hemoglobin is an independent risk factor for death [91]. Similar findings were also reported from two other studies from Singapore [92] and Tanzania [93]. All these findings confirm that interventions aimed at improving the nutritional status of HIV positive children are a relevant adjunct to ART.

Vaccines are among the greatest achievements of biomedical science and public health [94]. Unfortunately, vaccination is still perceived as unsafe and unnecessary by a growing number of individuals. Vaccine hesitancy is believed to be responsible for decreasing vaccine coverage and an increasing risk of vaccine-preventable disease outbreaks and epidemics [95]. In this contest, anti-vaccination campaigns have had a damaging impact on vaccine uptake. In order to achieve high vaccine coverage, all walls that impede vaccination including allergic reaction to a vaccine component or to a previous dose should be eliminated [96]. Moreover, some countries have introduced laws to require children to be vaccinated before school entry. Policies that mandate vaccination have always been controversial [97].

Bozzola et al. compared vaccination policies in children under 18 months against diphtheria, tetanus, pertussis, hepatitis B, poliovirus, *Haemophilus influenzae* type b, measles, mumps, rubella and varicella among different European countries [98]. They found that eleven Countries introduced mandatory vaccination and other recommended vaccinations. Latvia and Italy have 10 mandatory vaccines (tetanus, diphtheria, pertussis, *Haemophilus influenzae* type B, Hepatitis B, poliovirus, mumps, measles, rubella) in childhood. Other countries (i.e. Bulgaria, Croatia, Czech Republic, France, Hungary, Poland and Slovakia) have the same vaccines which are compulsory among children, except varicella. All European Countries recommended or introduced compulsory vaccinations for the following vaccinations: tetanus, diphtheria, pertussis, *Haemophilus influenzae* type B, hepatitis B, poliovirus, mumps, measles, rubella with the exception of Iceland that did not recommend hepatitis B vaccination. This study demonstrates that many European countries, including Italy, adopted compulsory policies for preventing the spread of infectious diseases and protecting the community, with encouraging results. Studies of the impact of mandatory immunization in high-, middle- and low-income countries in different contexts are urgently needed as many countries have enacted or are contemplating mandatory childhood immunization programs [99]. Regrettably in this scenario, there are still many differences between European countries as far as the type of vaccine used, number of doses and timing of vaccinations.

### Neonatology

#### 1 – electrolyte disturbances; 2 - neonatal intensive care; 3 - family integrated care

Birth asphyxia is defined as the presence of hypoxia, hypercapnia, and acidosis leading to systemic disturbances in newborns [100]. It is a common problem, with incidence from 0.5–2% of live births [101]. There is limited literature regarding electrolyte disturbances in asphyxiated newborn, despite they significantly affect perinatal morbidity, mortality and future management.

Thakur et al. have investigated electrolyte (sodium, potassium, calcium) disturbances in asphyxiated newborns of different severity [102]. They found that hyponatremia, hyperkalemia and hypocalcemia occurring in neonates with perinatal asphyxia may be responsible for increased morbidity and mortality and that hyponatremia and hyperkalemia are positively associated with the severity of birth asphyxia, thus confirming the data from Basu et al. [103]. In birth asphyxia the treatment of hyponatremia includes fluid restriction, rather than increased sodium load, till normalization of serum sodium are achieved. Correction of acidosis and use of potassium free fluid are the most useful measures to correct hyperkalemia, while serum potassium and electrocardiography (ECG) monitoring should be performed to avoid the complications associated to hyperkalemia.

Families satisfaction has become an important indicator of health care quality and patients’ opinions contribute to measure the quality of the delivered care [104–107]. In the Neonatal Intensive Care Units (NICU), the positive or negative experiences of parents may influence the lives of parents and infants over time and assessing NICU parents’ satisfaction is crucial to inform new directions for change [108–111]. Recently, a specific instrument, the EMpowerment of PArents in THe Intensive Care-Neonatology (EMPATHIC-N), has been developed in the Netherlands; it is a validated instrument which investigates different domains of care in NICUs from a family-centered care perspective [112]. Dall’Oglio et al. have recently translated, cultural adapted and validated the Dutch EMPATHIC-N instrument into Italian in order to provide a benchmark outcome measure [113]. They conducted a multi-center study in nine level III NICUs allocated across Italy. Parents were enrolled and received the EMPATHIC-N instrument on the day of discharge or within the first 3 days after the discharge of their child. Results demonstrate that the Italian version of the EMPATHIC-N has good psychometric properties, validity, and reliability showing and that, although the environment plays an important role for parents’ satisfaction, the behavior of the staff and the quality of parent-provider relationship still influence parents’ experience [114]. Other recent studies have confirmed that EMPATHIC-N is a valid and reliable measure for the assessment of parental satisfaction in the NICUs [112, 115, 116].

At present, the number of preterm births is rising. Preterm neonates are at an increased risk for a wide range of short- and long-term respiratory, infectious, metabolic and neurological complaints [117–119] and prematurity is also associated with high neonatal hospital costs, all of which decrease exponentially with increasing gestational age. The WHO proposed several relevant measures to improve the quality-of-life and health of preterm infants which include family-centered care. Family Integrated Care (FIC) is an approach that allows parents to provide non-medical routine care for their preterm infant during NICU hospitalization [120]. If greater involvement of parents in the care of their infant is wished, the NICUs need to be liberal in the visiting policies. Most NICUs in China have restricted visiting policies and parents have limited involvement in care. For this reason, He et al. conducted a pre-post intervention study at NICUs in two Chinese children’s hospitals including infants with BPD subdivided into a pre-intervention (*n* = 134; from December 2015 to September 2016) and a post-intervention (FIC) group (*n* = 115 neonates and their parents; from October 2016 to June 2017), with parents asked to be present and to care for their infant not less than 3 h per day [121]. The infants’ outcome measures were length-of-stay, breastfeeding, weight gain, respiratory and oxygen support, and parent hospital expenses. They demonstrated that the FIC model is feasible in NICUs and might result in significant improvements of infants’ clinical outcomes such as weight gain, breastfeeding time, breastfeeding rate and respiratory support time.

### Neurology

#### 1 – media device; 2 - cyberbullying; 3 - early-life seizures

Media devices (MD) are widespread today not only among adults, as also pre-school children are growing up in environments fool of internet connections, personal computer and video games. The percentage of children aged 0–8 years using a mobile device increased from 38% in 2011 to 72% in 2013 [122]. At present, almost all children use MD, most of kids start using a mobile MD before the age of 1 year, and a lot of them use a device daily, in particular smartphones and tablets [123]. Several studies have described the adverse effects of an early and prolonged exposure of pre-school children to digital technology, underlining the negative effects on the neurocognitive development, learning, well-being, sight and listening [124]. Bozzola et al. have investigated both beneficial and negative effects of media on pre-school children’s mental and physical health [125]. They found that touch screen usage may interfere with infant and toddler learning development. However, children younger than 3 years old can learn words through video if the experimenter/parent/caregiver provide additional verbal and non-verbal information during the live action sequences [126]. Moreover, mobile phones could reinforce what children are already learning at school. As far as children’s development, it is negatively affected by the television exposure [127], while drawing apps are appropriate for young children playing a positive role in their development [128]. MD inappropriate use is also associated to behavioral problems, obesity, sedentary, and physical discomfort, especially involving neck and shoulders [129]. Media usage may also interfere with sleep quality, in particular the presence of a television in the bedroom is associated with sleep terrors, nightmares and sleep talking [130]. Excessive smartphone use at a close reading distance might induce ocular fatigue, glare, and irritation and be responsible for acute exotropia [131]. Speech and language development may be compromised too. Difficulties in socializing, communicating and interacting with other kids and parents may be possible side effects [132]. Paediatrician and families should create a network to manage MD to minimize unhealthy habits and behaviors, avoiding negative effects on health, wellness, social and personal development [133]. Finally, in agreement with the American Academy of Pediatrics [123] and the Australian guidelines [134], media devices should not be used in children under 2 years of age. Media exposure should be limited to less than 1 h per day in children aged 2–5 years, to less than 2 h per day in children aged 5–8 years, to high-quality programming, just in presence of an adult, and to apps tested by a care-giver before the child usage.

Social media has had a profound effect on how children and adolescents interact. While there are many benefits to the use of social media, cyberbullying is now well recognized as a serious public health problem affecting children and adolescents [135]. It is defined as “any behavior performed through electronic or digital media by individuals or groups that repeatedly communicates hostile or aggressive messages intended to inflict harm or discomfort on others” [136]. Ferrara et al. have recently reviewed the data available in the literature about these rising phenomena [137]. Actually, 20 to 40% of children and adolescents have been victims of cyberbullying, with females and sexual minorities seemingly at higher risk. The most common methods for electronic bullying involve the use of instant messaging, chat rooms, and e-mail. Importantly, in most cases the electronic bully victims do not know the perpetrator’s identity, indeed anonymity, by promoting disinhibition, can lead to magnified aggression because the perpetrator may feel out of reach and immune to retribution. In this context, adolescents’ levels of social and emotional development leave them vulnerable to peer pressure and at the same time adolescents’ capacity to self-regulate is limited, generally they realize the severity of the situation after the effects have already occurred [138]. The motivation behind cyberbullying seems to be lack of confidence or the desire to feel better about themselves, a desire for control, finding it entertaining and retaliation, moreover there is a relation between cyberbullying and internet addiction. In cyberbullying, differently of traditional bullying, aggressive behaviors occur at any given time of the day, therefore the persistence of the bullying behaviors may result in even stronger negative outcomes than traditional bullying [136, 138]. Cyberbullying could lead to new onset psychological symptoms, somatic symptoms of unclear aetiology or a drop in academic performance. It seems to be that victims of cyberbullying have lower self-esteem, higher levels of depression, behavioral problems, substance abuse and experience significant life challenges [136, 138] which could result in suicidal behavior. Ferrara et al. between January 2011 and December 2013 identified 55 cases of suicide among Italian children and young adults aged less than18-years, and found that the second most frequent known cause of suicide is bullying [139]. In Italy, 2015 ISTAT data show that, among the media devices adolescent users, the 5.9% report being victims of cyberbullying [140]. As several suicides were linked to cyberbullying, the Italian Parliament has approved the so-called “anti-cyberbullying law” which makes it illegal to use the Internet to offend, slander, threaten or steal the identity of a minor, and ensure victims and their parents to demand that websites hosting abusive content is removed within 48 h [141]..

Seizures are not uncommon clinical manifestations in childhood. The term epilepsy defines the recurrences of two or more unprovoked seizures. Seizures starting in the first year of life including the neonatal period might have a favorable course, such as in infants presenting with Benign Familial Neonatal Epilepsy (BFNE), Febrile Seizures simplex (FSs) and Acute Symptomatic Seizures (ASS). However, in some cases, the onset of seizures at birth or in the first months of life have a dramatic evolution with severe cerebral impairment such as “epileptic encephalopathies”.

Pavone et al. provided an updated review of the conditions associated with seizures in the first year of life [142]. BFNE is a condition inherited as an autosomal dominant trait (mutation in the KCN gene, chromosome 20q13.33) and is characterized by seizures in the first days of life in otherwise healthy looking babies and are typically associated with a family history of neonatal seizures, neonatal prognosis is usually benign and the seizures tend to gradually disappear within the first months of life [143]. ASS is defined as a clinical seizure occurring at the time of a systemic insult or in close temporal association with a documented brain insult [144], they might follow trauma, intoxication, or anomalous administration of drugs or they could be induced by electrolytic dysregulation (acute hypoglycemia, hypocalcemia, and hyponatremia). The occurrence of the ASS is particularly high in the infantile period since, at this age the brain seems to be more susceptible to such insults. The seizures present most frequently as motor tonic-clonic generalized types and they usually have benign course. FS are the most common convulsive manifestations in childhood, FSs are defined as a short (< 15 min.) generalized seizures, not recurring within 24 h which occur during a febrile illness not resulting from an acute disease of the nervous system, in a child aged between 6 months and 5 years, with no neurologic deficits and no previous afebrile seizures [145]. Intravenous, intramuscular, buccal, intranasal or rectal benzodiazepines are administered to stop the crises while prophylactic pharmacologic treatment is not advised [146].

Febrile Seizures complex (FSc) are focal, or generalized, and prolonged seizures lasting more than 15 min, recurrence can happen within 24 h in the course of the same febrile episode, the temperature might not be elevated. Moreover, the crises might be associated with post-ictal neurologic abnormalities, most frequently post-ictal palsy, or manifest in subjects with previous neurologic deficits. They might be present in alternation with afebrile seizures, or in members of a family affected by Genetic Epilepsy with Febrile Seizures plus (GEFS+, autosomal dominant disorder characterized by mutations of the gene SCN2A or less frequently of SCN1B) [147]. Febrile status epilepticus might also be recorded. The acute treatment is based on the use of benzodiazepines. In FSc, prophylactic treatment might be useful in reducing the frequency and the duration of the crises but is not considered able to prevent the onset of subsequent epileptic seizures [148].

### Nutrition

#### 1– obesity; 2 – dietary habits; 3 – malnutrition

Obesity is a major problem in Western Societies. The position statement on obesity in children and adolescents of Italian Society of Pediatrics stated the diagnostic criteria for defining obesity according to World Health Organization reference curves and ascribed the specific causes of secondary obesity (endocrine, hypothalamic, genetic, iatrogenic) [149]. Moreover, obesity comorbidities have been evaluated, such as hypertension by the definition of the blood pressure values; dyslipidemia with the indication for the oral glucose tolerance test in children and adolescents with overweight or obesity; polycystic ovary syndrome; gastrointestinal, renal, orthopedic or respiratory complication. Dietary advices have been fulfilled showing a variety of dietary regimens associated to physical exercise. The indicators of successful treatment are the BMI standard deviation scores, and in case of low weight loss, other behavioral indicators. If the lifestyle-based intervention, pharmacological treatment or surgery should be used, including orlistat that represents the only drug available for children and bariatric surgery for adolescents with severe obesity resistant to all other therapies.

Subcutaneous adiposity is correlated with major health diseases and reduced life expectancy [150]. Visceral fat accumulation is generally associated with insulin resistance and hyperglycemia, dyslipidemia and hypertension, all together called metabolic syndrome. This syndrome occurs in 31% of obese patients and it is related to type 2 diabetes, coronary heart disease and cerebrovascular disorders, named “noncommunicable diseases”.

The pre-natal period since conception and furthermore the whole first year of life are crucial for controlling obesity risk in the life, so it is important to promote a healthy lifestyle since this period called “the first 1000 days of life” [151]. Beneficial and possibly beneficial interventions during pregnancy aimed at reducing childhood obesity are: balanced energy/protein supplements, low glycemic index dietary recommendations, dietary counseling alone or supported by physical activity or supervised exercise. An alternative approach to weaning, named baby-led weaning has been proposed for preventing obesity. Baby-led weaning [152] consists in offering to infants directly whole pieces of food that put themselves in their mouth during family mealtimes from 6 month of age. In this way the child himself can decide and control the quality and the quantity of food and the speed to eat. It may promote the preference of a larger choices of food because of the distinctive tastes of proposed foods. Moreover, it can be in line with the recent recommendations of the “early” introduction (after 4 months of age and before 12 months) of all foods, including allergenic ones, to prevent the development of food allergies [153]. However, there is an augmented risk of choking and gagging, because there are variations in age of babies for developing the skills of chewing, biting and swallowing [154]. This means that frequently babies continue milk formulas with the risk of inadequate iron and energy intake and growth faltering. Regarding prevention of obesity onset, no difference in Body Mass Index has been found at 24 months [155].

A three-day record about the nutrient’s intake has been composed by parents of 443 Italian children during the first year of life (age 6.4–131 months) [36]. Interestingly, most of children exceeded in the doses of protein intake, simple carbohydrates and saturated fats above the limits of the Italian Dietary Reference Values, with a low intake of fiber and polyunsaturated fats. Regarding micronutrients, the vitamin D intake was very low in all age groups. Early interventions to support healthy lifestyle habits are essential to ensure long term healthy outcomes. To reduce the risk of noncommunicable diseases several healthy advices during the first years of life have been shown significant, such as breastfeeding, using lower protein formulas for formula-fed infants, increasing fruit and vegetables and reducing junk foods and sugar-sweetened drinks [156]. With as much importance, physical activity interventions since peri-conception and pregnancy period to childhood are efficacious to reduce the risk of non-communicable diseases in childhood [157].

Also developing countries are affected by overweight/obesity. Particularly, in Ethiopia overweight and/or obesity are frequent among school-aged children, revealing a prevalence of 11.9% in a study from Bahir Dar City [158]. Risk factors for obesity were higher wealth status, dietary habits (fast food intake, low fruit and vegetable use), and sedentary lifestyle.

From the other side of the coin, in the developing countries acute severe malnutrition represent a major health burden [159]. Several indicators have been identified to define the risk of malnutrition in childhood. Childhood acute malnutrition is linked with the number of children in the household, unprotected drinking water sources, latrine availability, hand washing practice before food preparation and child feeding, childhood diarrheal disease, and number of vaccinations [160]. In fact, repeated diarrhea attacks and infections leads to weight loss and compromise a child’s nutritional status. Moreover, the mother’s age at birth, birth interval, socioeconomic status, father’s educational level and starting complementary feeding at the age of 6 months are important determinants of severe acute malnutrition among children from Nepal [161].

Interventions to prevent malnutrition have been developed. A supportive supervision trial has been tested with the aim of achieving higher adherence to guidelines and good quality of care at outpatient level for malnourished children [162]. This approach has been shown efficacious in terms of cure rate and correctness in complementary treatment in six health centers of Uganda. In Southern Ethiopia School Feeding Program [163] showed positive effects on the mean BMI-for-age, significant increase of height-for-age and improvements in the dietary diversity and class attendance.

### Surgery

#### 1 - day surgery procedures 2 - esophageal atresia

The Italian Society of Pediatric Surgery (SICP) and The Italian Society of Pediatric Anesthesia (SARNePI) produced EBM-guidelines on the feasibility of day surgery in relation to different pediatric surgical procedures [164]. Children represent an interesting model for day-case management since they commonly have few co-morbidities and most pediatric operations are procedures suitable for day surgery. The main aspects of the pre-operative assessment involve clinical factor with structured questionnaires and clinical evaluation, socio-familiar factors to check a parental/environmental adequacy, surgical factors for appropriacy of operations and discharge. An assessment of the pediatric operations in day surgery, including enrollment, anesthesia and discharge, have been reported. One of the most common ambulatory surgical procedures in pediatric age is tonsillectomy with or without adenoidectomy, since it represents the first-line treatment for patients with obstructive sleep-disordered breathing (oSDB). Ambulatory tonsillectomies in the United States in 2010 have been estimated 339,000 in children (median age 7.8 years) [165]. Inpatient procedures were associated with younger age (< 3 years) and patients affected by oSDB. Perioperative events such as apnea, hypoxia, or bleeding occurred 7.8% of cases.

The prescription of drugs for analgesia after surgical procedures warrant careful consideration also because they can elicit anaphylactic reactions [166]. In particular, the use of opioids in pediatric population after surgical procedures still needs standardization. A retrospective study observed that opioids were prescribed in 37.4% of cases on discharge after 329 pediatric ambulatory hernia surgery procedures [167]. Doses varied from 0 to a maximum of 33 doses between surgeons for the same procedure. Moreover, an increased probability of opioid prescription was associated with increasing patient age with a prevalence of opioids prescription of 1.5% among children under 1 year of age and 91.6% in children aged 15 to 17 years.

Esophageal atresia (EA) is an anatomic variant that may include a tracheoesophageal fistula and be correlated to the complete stenosis of the esophagus. A recent study recruited 67 newborns with EA classified according to different clinical presentations: isolated EA; EA plus a concomitant single malformation; VACTERL association; and EA in the context of multiple congenital anomalies [168]. Many evolution patterns have been considered according to the mortality, the duration of the total parental nutrition, the invasive mechanical ventilation, or the corrected gestational age at discharge. The most complex conditions were associated with higher levels of intensive care.

A recent review of collected databases included 207 newborns with EA and/or tracheoesophageal fistula (TEF) from 2008 to 2017 and showed the association of laryngotracheal abnormalities (LTA) in 40% of cases [169]. All patients underwent to flexible laryngotracheoscopy in order to analyze the impact of airway anomalies. Infants with EA/TEF and LTA were characterized by higher frequency of tracheostomy and increased mortality rate compared with those without LTA.

Severe tracheomalacia is frequently associated to EA/TEF and characterized by dynamic airway collapse. It needs to be treated with surgery at the time of initial EA repair (primary treatment) or secondary treatment. A retrospective review collected 118 patients with EA who underwent posterior tracheopexy in Boston Children’s Hospital for 4 years, showing that this procedure has significant improvements in clinical symptoms and degree of airway collapse on bronchoscopy [170]. Long-term quality of life in patient with EA is often characterized by physical limitations since the survival is increasing over the last years. Surprisingly, among 17 German patients with EA aged 3.12 years, 93% of cases participated regularly to physical education classes at school and 86% of them in fitness events [171].

## Conclusion

Last year, investigations have offered great progresses in recognizing the pathomechanisms of pediatric diseases and has unraveled different beneficial approaches. The development of novel therapies has allowed not only an improved disease management but also a change of the natural history of diseases. Intensive attempts have been made to identify risk factors that can be modified to prevent the diseases. However, there are several unmet needs, for example in understanding interaction between genes and environment, better strategies for diagnostics, response to treatment according to disease severity, and primary prevention.

## Data Availability

Data sharing not applicable to this article as no datasets were generated or analyzed during the current study.

## Abbreviations

ART: Anti-retroviral therapy
ASD: Autism spectrum disorder
ASS: Acute Symptomatic Seizures
BFNE: Benign Familial Neonatal Epilepsy
CAA: Coronary artery aneurysms
CAH: Congenital adrenal hyperplasia
EA: Esophageal atresia
ECG: Electrocardiography
ED: Emergency department
EMPATHIC-N: EMpowerment of PArents in THe Intensive Care-Neonatology
ENT: Ear, nose, and throat
FGIDs: Functional gastrointestinal disorders
FIC: Family Integrated Care (FIC)
FSc: Febrile Seizures complex
FSs: Febrile Seizures simplex
GAGs: Glycosaminoglycans
GEFS+: Genetic Epilepsy with Febrile Seizures plus
HIV: Human immunodeficiency virus
IBS: Irritable bowel syndrome
IMDs: Inherited metabolic disorders
IVIG: Intravenous immunoglobulin
KD: Kawasaki disease
LTA: Laryngotracheal abnormalities
MD: Media devices
MPS: Mucopolysaccharidoses
NBS: Newborn screening
NGS: Next-generation sequencing
NICU: Neonatal Intensive Care Units
OSA: Obstructive sleep apnoea
oSDB: obstructive sleep-disordered breathing (oSDB)
SARNePI: Italian Society of Pediatric Anesthesia
SH: Subclinical hypothyroidism
SICP: Italian Society of Pediatric Surgery (SICP)
SOR: Standard oral rehydration
TEF: Tracheoesophageal fistula
VKDB: Vitamin K deficiency bleeding
